# Sequential Recovery of Heavy and Noble Metals by Mussel-Inspired Polydopamine-Polyethyleneimine Conjugated Polyurethane Composite Bearing Dithiocarbamate Moieties

**DOI:** 10.3390/polym11071125

**Published:** 2019-07-02

**Authors:** Dingshuai Xue, Ting Li, Guoju Chen, Yanhong Liu, Danping Zhang, Qian Guo, Jujie Guo, Yueheng Yang, Jiefang Sun, Benxun Su, Lei Sun, Bing Shao

**Affiliations:** 1 State Key Laboratory of Lithospheric Evolution, Institute of Geology and Geophysics, Chinese Academy of Sciences, Beijing 100029, China; 2 School of Public Health, Capital Medical University, Beijing 100069, China; 3 State Key Laboratory for Comprehensive Utilization of Nickel and Cobalt Resources, Jinchang 737100, China; 4 Beijing Key Laboratory of Diagnostic and Traceability Technologies for Food poisoning, Beijing Center for Disease Prevention and Control, Beijing 100013, China; 5 Center for Biological Imaging, Institute of Biophysics, Chinese Academy of Sciences, Beijing 100101, China

**Keywords:** polyurethane, graphene, metallurgy, polymeric composites, thiocarbamate

## Abstract

Dithiocarbamate-grafted polyurethane (PU) composites were synthesized by anchoring dithiocarbamate (DTC) as a chelating agent to the polyethyleneimine-polydopamine (PE-DA)-functionalized graphene-based PU matrix (PE-DA@GB@PU), as a new adsorbent material for the recovery of Cu^2+^, Pb^2+^, and Cd^2+^ from industrial effluents. After leaching with acidic media to recover Cu^2+^, Pb^2+^, and Cd^2+^, dithiocarbamate-grafted PE-DA@GB@PU (DTC-g-PE-DA@GB@PU) was decomposed and PE-DA@GP was regenerated. The latter was used to recover Pd^2+^, Pt^4+^, and Au^3+^ from the copper leaching residue and anode slime. The present DTC-g-PE-DA@GB@PU and PE-DA@GB@PU composites show high adsorption performance, effective separation, and quick adsorption of the target ions. The morphologies of the composites were studied by scanning electron microscopy and their structures were investigated by Fourier transform infrared (FT-IR) spectroscopy and Raman spectroscopy. The effects of pH values, contact time, and initial metal ion concentration conditions were also studied. An adsorption mechanism was proposed and discussed in terms of the FT-IR results.

## 1. Introduction

Heavy metal ions (Cu^2+^, Pb^2+^, and Cd^2+^) are non-biodegradable, accumulative, and persistent environmental contaminants. In recent years, the surge of industrial effluents and mine wastewater has intensified environmental problems, posing a severe harm to ecological and global public health because of the carcinogenicity, high toxicity, and biological accumulation of such ions [1]. Pd^2+^, Pt^4+^, and Au^3+^ are well-known noble metals (NMs) used for coins, jewelry, catalysts, and electric and corrosion-resistant materials. As NM resources are limited, their demand in the jewelry market and for medical applications is generally quite high [2]. Consequently, considerable research efforts have been made toward the removal of heavy metal ions from industrial wastewater and recovery of NMs from industrial waste, which usually involve using adsorbents derived from polyurethane (PU) by virtue of its extremely low cost, 3D porous structure, easy handling and storage [3]. Jinchuan Group Ltd. is China’s biggest platinum group metals producer. During the electrorefining process, some heavy metals (HMs) in industrial effluents are discharged to the tailings dam and some NMs accumulate in the byproduct copper leaching residue and anode slime. The latter is an important source for recycling and recovery of Au, Pd, and Pt [4]. These sludges are categorized as hazardous wastes, and their storage and disposal are very costly. However, as these wastes contain valuable metals in abundance, it is extremely important to maximize the utilization and recycling of HMs and NMs economically to achieve both environmental protection and sustainable development.

For this purpose, many kind of adsorbents have been synthesized and various techniques such as nanoparticles [5], nanofibers [6,7], ion exchange [8,9], membrane separation [10,11], and solvent extraction [12,13] have been investigated over the past few years. However, the extraction of HM and NM ions from wastes is still challenging. In this respect, the critical issues are low selectivity and insufficient adsorption capacity of the adsorbents. PU is the most practical adsorbent material for NMs. Graphene-based composite materials have attracted considerable attention because of the large specific surface area of graphene [14]. It has been shown that the fusion of graphene oxide (GO) with PU polymers can enhance the mechanical properties and surface area of graphene-based PU (GB@PU) [15].

In the present work, a polyethyleneimine-polydopamine (PE-PDA) hybrid coating was spontaneously co-deposited onto the surface of GB@PU [16]. By taking advantage of the self-polymerization of catechol-inspired natural PDA, PE-DA coated GB@PU (PE-DA@GB@PU) hybrid was prepared via Michael addition or Schiff base reactions [17]. The homogeneous and well-controlled coating layer of PDA on GB@PU improved the hydrophilicity of the GO sheets. Simultaneously, the PDA layer offered abundant active sites to graft the high-density PE molecule. The prepared PE-DA@GB@PU composite exhibited excellent selectivity and high adsorption capacity for NMs. In order to further improve the adsorbent performance, dithiocarbamate (DTC) was anchored on the surface of PE-DA@GB@PU. DTC could strongly chelate with various metal ions owing to its sulfur and nitrogen groups, which provided lone pair electrons form chemical bonding [18,19]. First, DTC-g-PE-DA@GB@PU was used to recover HMs from industrial effluents. When the adsorption efficiency of DTC-g-PE-DA@GB@PU decreased, 0.1 M HCl was used to leach the HMs and regenerate PE-DA@GB@PU, which was used to recover NMs from the copper leaching residue and anode slime of Jinchuan Group Ltd. This new design strategy greatly improves sorbent utilization efficiency and saves the raw material. Continuous recycling of two different types of metal ions is more environmentally-friendly than other sorbents. In addition, the adsorbents are readily regenerated and exhibit excellent stability and recyclability.

## 2. Materials and Methods

### 2.1. Materials

Open-cell type polyether PU was purchased from Taobao (China). Graphene oxide solution was acquired from XFNANO Materials Tech Co., Ltd. (China). Dopamine hydrochloride (DA) and PE (*M*_w_ = 600 Da) were obtained from Sigma-Aldrich (USA) and Aladdin (China), respectively. Other chemicals, including dopamine hydrochloride (DA), PE (*M*_w_ = 600 Da), ethanol (EtOH), sodium hydroxide (NaOH), ammonium hydroxide, and carbon disulfide (CS_2_), were obtained from Sinopharm Chemical Reagent Co., Ltd. The stock standard solutions for Cu^2+^, Pb^2+^, Cd^2+^, Pd^2+^, Pt^4+^, and Au^3+^ were procured from Spex CertiPrep Inc. (Metuchen, NJ, USA).

### 2.2. Instrumentation

An inductively coupled plasma optical emission spectrometer (ICP-OES) was used to determine the concentration of metal ions (Table S1, Supplementary Materials). A 540 Zeiss scanning electron microscope (SEM, Merlin, Oberkochen, Germany) with energy dispersive X-ray spectroscopy (EDS, Oxford Instruments, Oxford, the UK), Fourier-transform infrared spectrometer (FTIR, Bruker VERTEX 70v, Karlsruhe, Germany), Raman spectrometer (HR800, HORIBA Jobin Yvon, Paris, France) and wide-angle X-ray diffraction (WAXD, Panalytical X’Pert PRO, Almelo, The Netherlands) were used for the characterization of the new materials.

### 2.3. Synthesis of PE-DA@GB@PU and DTC-g-PE-DA@GB@PU

**Scheme 1:**
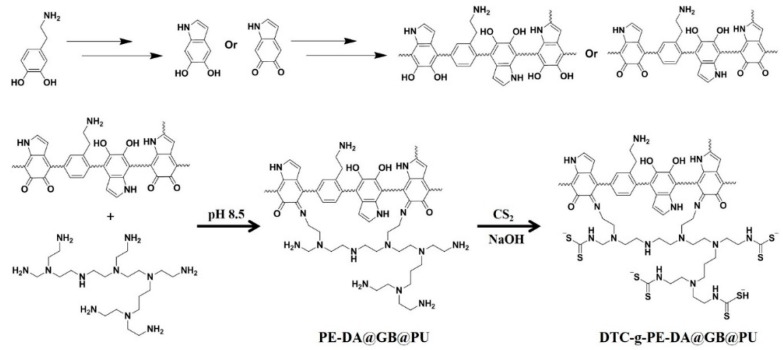
Synthetic routes of PE-DA@GB@PU and DTC-g-PE-DA@GB@PU.

The PE-DA@GB@PU and DTC-g-PE-DA@GB@PU materials were prepared from PU, as illustrated in Scheme 1. Raw PU (3.0 g) was boiled in 2 M HCl solution for 2 h for the amination of PU (APU). GB@PU was prepared by a dip coating and solvothermal synthesis method [20,21]. First, AP was immersed in GO (2.0 mg mL^−1^) solution, then transferred into a Tetrafluoro autoclave. After ultrasonication for 1 h, the Tetrafluoro autoclave was heated to 90 °C and maintained at this temperature for 12 h. Next, GB@PU was dried in a vacuum oven at 50 °C. Finally, GB@PU was immersed in Tris buffer solution (pH = 8.5) of DA (2 mg/mL) and PE (4 mg/mL) in a mass ratio of 1:1 (25 °C, 24 h). After modification, the samples were withdrawn and washed several times using deionized water, then dried in a vacuum oven at 50 °C overnight. Subsequently, PE-DA@GB@PU was immersed in 20 mL of 1.0 M NaOH solution, and 10 mL of 0.1 M CS_2_ solution in ethanol was added dropwise to the reaction system. The reaction mixture was then stirred at room temperature for 24 h. Finally, DTC-g-PE-DA@GB@PU was filtered and rinsed four times with water [22].

### 2.4. Static Adsorption Experiments

Adsorption experiments were carried out by batch method. A series of standards or sample solutions containing Cu^2+^, Pb^2+^, Cd^2+^, Pd^2+^, Pt^4+^, and Au^3+^ were transferred into a 50-mL triangular bottle with a grinding plug. The pH of each solution was adjusted to the desired value and the volume was adjusted to 20 mL. Either PE-DA@GB@PU or DTC-g-PE-DA@GB@PU (0.02 g) was added, and the mixture was shaken vigorously to attain equilibrium by a vibrating machine. The remaining metal ions in the solution were determined by ICP-OES. The amounts of HM and NM ions adsorbed by the adsorbent were calculated by the difference.

The percentage of each metal ion adsorbed on the adsorbent (*A*) was calculated according to Equation (1):

(1) A=(Di−De)∗100%Di

After equilibration, the extent of adsorption (F_e_ (mg g^−1^)) was calculated according to Equation (2):

(2) Fe=(Di−De)Bw

where *D*_i_ and *D*_e_ are the initial and equilibrium concentrations of the metal ions in the solution, respectively; and *B* and *w* are the volume (mL) and mass (g) of the solution and adsorbent, respectively.

## 3. Results

Dopamine can easily form insoluble PDA in alkaline solutions via versatile reactions with molecules containing thiol or amine groups by Michael addition and/or Schiff base reactions. Herein, PE was introduced on the GB@PU surface with PE-DA hybrid coating because it had a larger amine density, which proved advantageous for interaction with CS_2_ to form dithiocarbamate composites. The reaction scheme is represented in Scheme 1 [23,24]. 

### 3.1. Characterization of PE-DA@GB@PU and DTC-g-PE-DA@GB@PU

**Figure 1:**
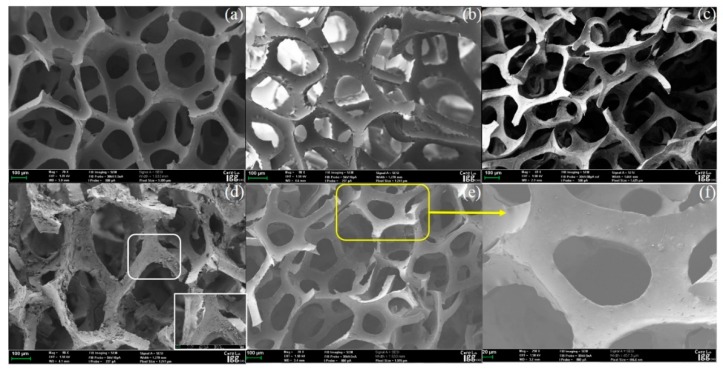
SEM image of (**a**) raw PU, (**b**) APU, (**c**) GB@PU, (**d**) PE-DA@GB@PU, and (**e**) DTC-g-PE-DA@GB@PU, (**f**) magnification SEM image of (**e**).

Raw PU (Figure 1a) at 70× magnification exhibited a smooth reticular skeleton, whereas the surface of the APU was denuded (Figure 1b). As shown in Figure 1c, the appearance of GB@PU was markedly different from that of raw PU and APU. Raman scattering revealed that the coating observed on the skeleton of GB@PU was composed of graphene sheets (Figure S1, Supplementary Materials). The skeleton of PE-DA@GB@PU was covered with a large number of microscale protrusions (Figure 1d). As shown in Figure 1e,f the skeleton of DTC-g-PE-DA@GB@PU was more smooth without protrusions as compared with PE-DA@GB@PU.

**Figure 2:**
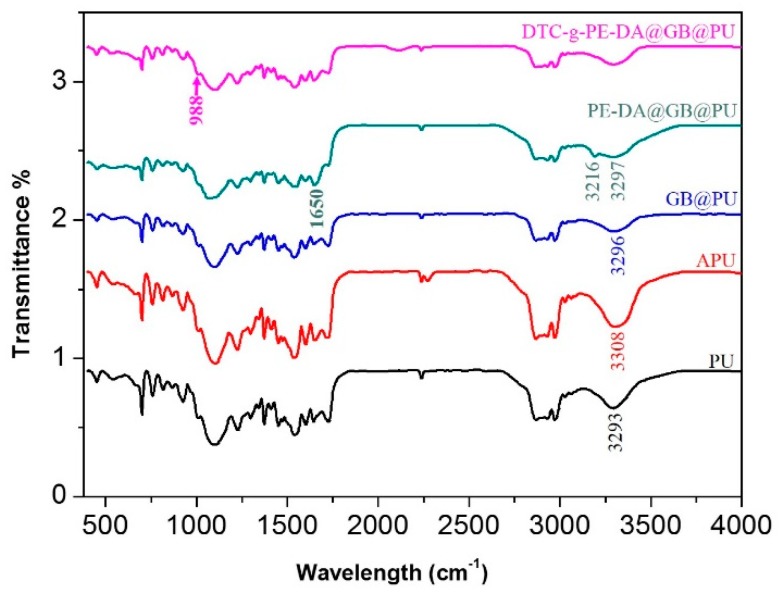
FTIR spectra of (**a**) raw PU, (**b**) APU, (**c**) GB@PU, (**d**) PE-DA@GB@PU, and (**e**) DTC-g-PE-DA@GB@PU.

The chemical structures of PU, APU, GB@PU, PE-DA@GB@PU and DTC-g-PE-DA@GB@PU were analyzed by FTIR spectroscopy, and their FTIR spectra are shown in Figure 2. The broadening of the band at 3308 cm^−1^ in the spectrum of APU was ascribed to stretching vibration of N–H for PU shifted to 3293 cm^−1^ for APU [25]. After graphene coated on the APU skeleton, the peaks of GB@PU between 2700 and 3500 cm^−1^ became weaker, because the graphene coating inhibited spectral absorption. Compared to APU and GB@PU, PE-DA@GB@PU showed a new absorption peak at ~1650 cm^−1^ and a broad band from 3200 to 3600 cm^−1^. The former could be assigned to the carbonyl groups derived from dopamine oxides, whereas the latter likely corresponded to the stretching vibration of hydroxy or amino groups of dopamine and PE [17]. For DTC-g-PE-DA@GB@PU, the characteristic peak of NH_2_ group at 3216 cm^−1^ disappeared and a new adsorption band could be observed at 958 cm^−1^, which indicated that the amino groups had reacted with CS_2_. However, the characteristic peaks of the DTC group (N–CS_2_ vibration and C=S vibration) were obscured by the strong absorption peaks of the PU matrix at ~1490 and 1100 cm^−1^. 

### 3.2. pH Effect

**Figure 3:**
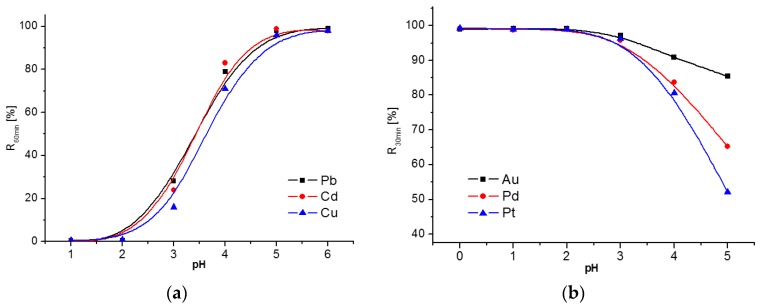
Effect of pH on the adsorption of Cu^2+^, Pb^2+^, and Cd^2+^ monometallic solutions (10.0 μg mL^−1^) by DTC-g-PE-DA@GB@PU (**a**) and the adsorption of Pd^2+^, Pt^4+^, and Au^3+^ monometallic solutions (1.0 μg mL^−1^) by PE-DA@GB@PU (**b**). Other conditions: adsorbent, 20 mg; shaking time: heavy metal ions 60 min, noble metal ions 30 min; and temperature, 25 °C.

Solution pH is one of the most important factors for the metal ions adsorption [26]. The influence of pH on the sorption of HM and NM ions on DTC-g-PE-DA@GB@PU and PE-DA@GB@PU, respectively, was studied over a pH range (Figure 3a,b). As shown in Figure 3a, there was a general increase in the recoveries of these ions with increasing pH of the solution. At low pH, the H^+^ ions competed with HM ions for the ion-exchange reaction at the adsorption sites, and accordingly, low adsorption recoveries were observed. With increasing pH, the concentration of H^+^ ions decreased and the active adsorption sites mainly turned into dissociated states, which were sufficient for catching the HMs by coordination interaction between dithiocarbamate groups and HM ions. According to the literature [27], DTC compounds are generally decomposed into the constituent amine and carbon disulfide in acidic media (presented in Figure S2, Supplementary Materials). After 0.1 M HCl was used to elute the HM ions from DTC-g-PE-DA@GB@PU, PE-DA@GB@PU is regenerated. PE-DA@GB@PU exhibited excellent extraction yields of Pd^2+^, Pt^4+^, and Au^3+^ over a wide pH range because of the rich amino groups and flexible chains of PE [17] (Figure 3b).

### 3.3. Adsorption Isotherms

**Figure 4:**
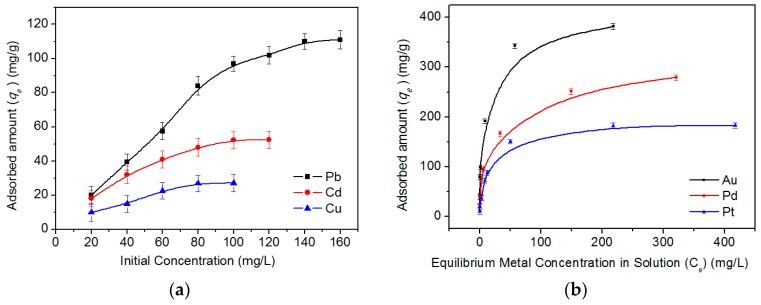
Adsorption isotherms of Cu^2+^, Pb^2+^, and Cd^2+^ onto DTC-g-PE-DA@GB@PU (**a**) and Pd^2+^, Pt^4+^, and Au^3+^ onto PE-DA@GB@PU (**b**). Other conditions: solution pH = 6 for DTC-g-PE-DA@GB@PU, solution pH = 0 for PE-DA@GB@PU; volume, 20 mL; adsorbent dose, 20 mg; contact time: 30 min for PE-DA@GB@PU; 60 min for DTC-g-PE-DA@GB@PU, temperature, 25 °C; *n* = 3.

The adsorption capacities of DTC-g-PE-DA@GB@PU for HM ions and PE-DA@GB@PU for NM ions were assessed using the equilibrium adsorption isotherm by varying the initial concentrations (The concentrations of Cu, Pb, Cd, Pd, Pt, and Au were in the ranges 20–100, 20–180_,_ 20–120, 20–800, 20–600, and 20–800 μg mL^−1^, respectively), as shown in Figure 4.

The equations of Langmuir, Freundlich, and Dubinin–Radushkevich isotherm models were used to analyze the experimental data. The experimental parameters are presented in Table 1. 

The adsorption isotherms of Pb^2+^, Cd^2+^, Pd^2+^, Pt^4+^, and Au^3+^ fitted well with the Langmuir model, which implied monolayer adsorption (linear correlation coefficients values (R^2^) for this model were greater than 0.9935). The theoretical F_max_ values of Pb^2+^, Cd^2+^, Pd^2+^, Pt^4+^, and Au^3+^ (113.9, 57.1, 285.7, 185.2, and 384.6 mg g^−1^, respectively) acquired from the Langmuir model were close to the experimental values (111.9, 53.8, 279.6, 175.3, and 380.5, mg g^−1^, respectively) at 25 °C. As for Cu(II), the D–R isotherm model fitted best to the experimental data, as concluded when its R^2^ value was compared with the R^2^ values of the Langmuir and Freundlich models. In addition, the maximum adsorption Q_D-R_ value calculated from the experimental data (30.6 mg g^−1^) was in good accordance with the experimental Q value (28.7 mg g^−1^) (Figures S3–S5, Supplementary Materials). Comparison of the maximum capacities of DTC-g-PE-DA@GB@PU and PE-DA@GB@PU with those of other adsorbents is shown in Table S2 (Supplementary Materials). Shannon reported [28] that the ionic radii of the heavy metal ions are in the following order: Pb^2+^ > Cd^2+^ > Cu^2+^. As is all known, dithiocarbamate group can bond to metal ions through its sulfur moiety. Based on the hard and soft acids and bases (HSAB) principle, sulfur is a soft base, which easily forms bonds with large, highly polarizable metal ions (soft-soft interaction). For the three HM ions, Pb^2+^ is much larger and much softer (more polarizable) than the others and interacts more strongly with sulfur. As metal ions become larger and more polarizable (Cu^2+^ < Cd^2+^ < Pb^2+^), the tendency for attachment of the increasingly “soft” metal ions to sulfur increases. The resultant [Pb(DTC)_4_]^2−^ ions is a soft–soft combination, while [Cu(DTC)_4_]^2−^ is a relatively hard–soft combination, consistent with the HSAB prediction. Therefore, the maximum adsorption capacity order is Pb^2+^ > Cd^2+^ > Cu^2+^. Meanwhile, the adsorption rate has a similar trend. In fact, other researchers have reported the same trend as that obtained in our study [27,29,30].

### 3.4. Adsorption Kinetics

**Figure 5:**
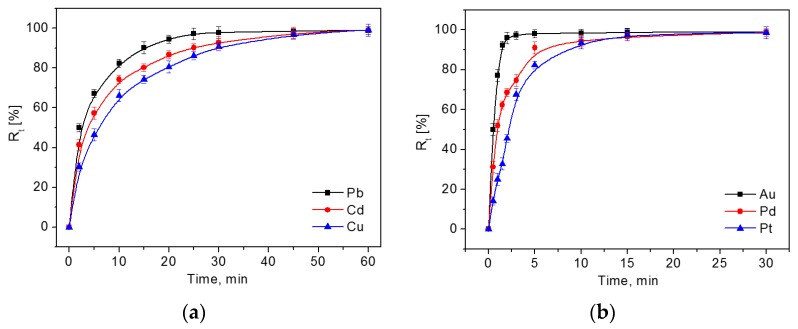
Effect of contact time on the adsorption of 10.0 μg mL^−1^ Cu^2+^, Pb^2+^, and Cd^2+^ onto DTC-g-PE-DA@GB@PU (**a**) and 1.0 μg mL^−1^ Pd^2+^, Pt^4+^, and Au^3+^ onto PE-DA@GB@PU (**b**). Other conditions: solution pH = 6 for DTC-g-PE-DA@GB@PU, solution pH = 0 for PE-DA@GB@PU; volume, 20 mL; adsorbent dose, 20 mg; temperature, 25 °C; (*n* = 3).

Figure 5 illustrates the adsorption of HM (Figure 5a) and NM (Figure 5b) ions on the adsorbent as a function of time. The adsorption plots showed that the initial adsorption rate of HM and NM ions was high. With the passage of time, the rate of percent adsorption decreased and then reached equilibrium, which was due to the gradual occupation of the available adsorption sites on DTC-g-PE-DA@GB@PU and PE-DA@GB@PU.

The results of various adsorption parameters obtained from the kinetic models are presented in Table 2. The pseudo-second-order kinetic model fitted well as compared to the pseudo-first-order model. The plots were not linear over the time range, implying that more than one process affected the adsorption process, thereby confirming that adsorption involved multiple stages (Figures S6–S8, Supplementary Materials).

### 3.5. Adsorption Mechanism

In order to understand the mechanism of adsorption of Cu^2+^, Pb^2+^, and Cd^2+^ onto DTC-g-PE-DA@GB@PU, and Pd^2+^, Pt^4+^, and Au^3+^ onto PE-DA@GB@PU, FTIR spectroscopy studies were conducted for DTC-g-PE-DA@GB@PU and HM ions loaded DTC-g-PE-DA@GB@PU (abbreviated as DTC-g-H), and PE-DA@GB@PU and NM ions loaded PE-DA@GB@PU (abbreviated as PE-N).

**Figure 6:**
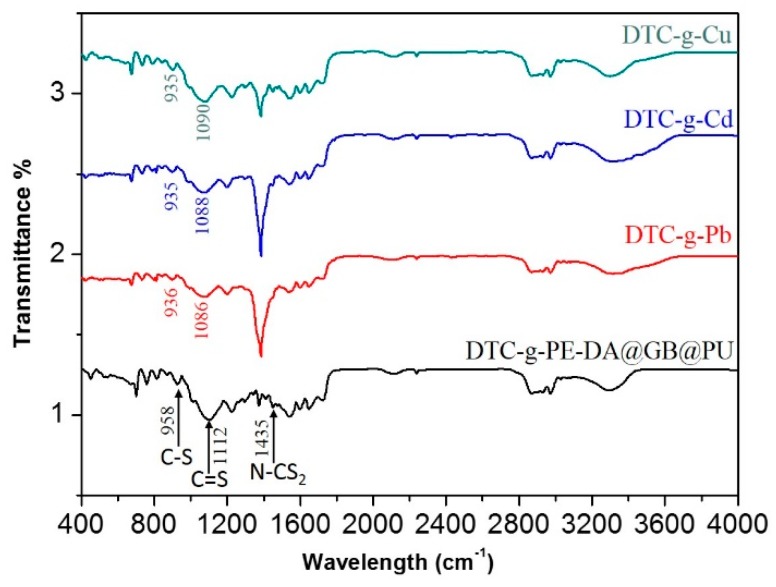
FT-IR spectra of DTC-g-PE-DA@GB@PU adsorbent and the DTC-g-Cu, DTC-g-Pb, and DTC-g-Cd complexes.

The FT-IR spectrum for DTC-g-PE-DA@GB@PU (Figure 6) showed peaks at 1112, 958, and 1435 cm^−1^, which were attributed to C=S vibration, C–S stretching vibration, and N–CS_2_ vibration, respectively [19]. There was a red-shift and decrease in intensity of the characteristic FTIR DTC-g-H signals (C–S and C=S stretching vibrations) after the adsorption of HM ions. In addition, sharp new peaks were observed at 1375 cm^−1^ in the spectra of DTC-g-Cu, DTC-g-Pb, and DTC-g-Cd. These results indicated that a strong metal-ligand bond had formed between the metal ions and chelating groups in DTC-g-PE-DA@GB@PU.

**Figure 7:**
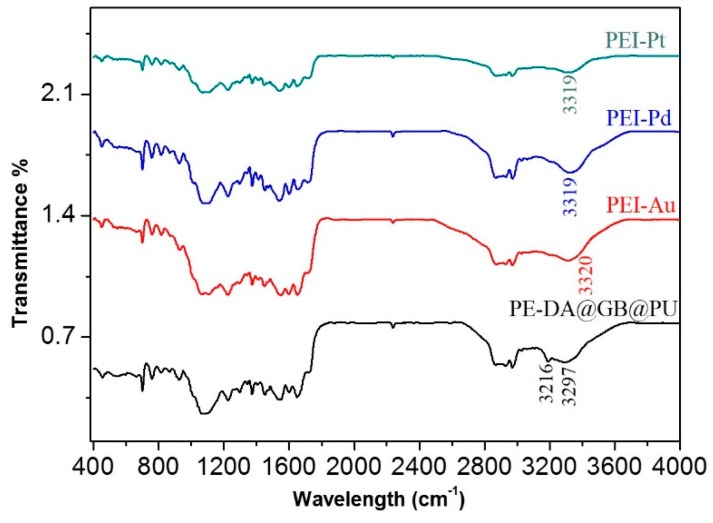
FT-IR spectra of PE-DA@GB@PU adsorbent and the PE-Pd, PE-Pt and PE-Au complexes.

As seen in Figure 7, after the adsorption of NM ions, the N–H stretching peak shifted from 3297 to 3319 cm^−1^ and the peak 3216 cm^−1^ disappeared altogether, which was likely attributable to either the electrostatic attraction between the positively charged amine groups in the adsorbent and the negatively charged NM anions [31,32], or because the nitrogen atoms in the amino groups coordinated with the NM ions. Moreover, the interaction between the NM ions and NH group resulted in the broadening of the peaks from 3100 to 3600 cm^−1^.

**Figure 8:**
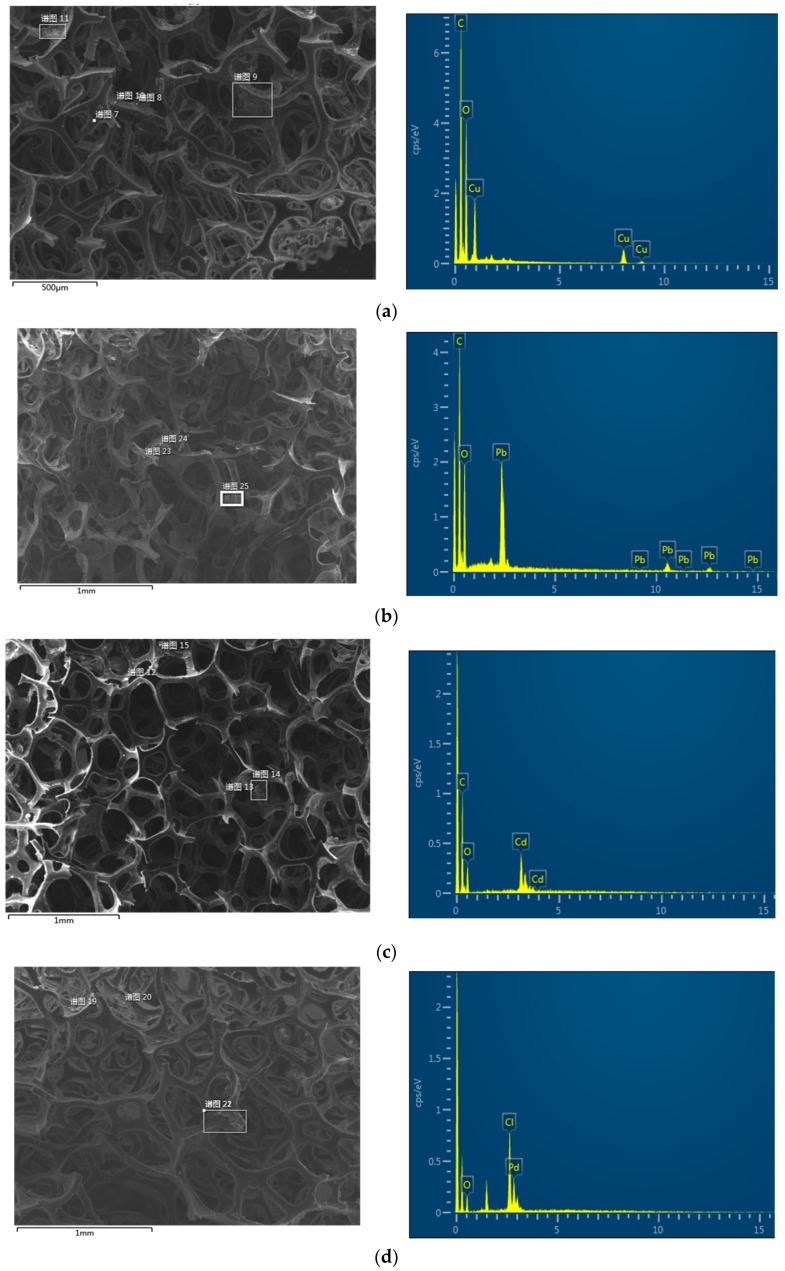

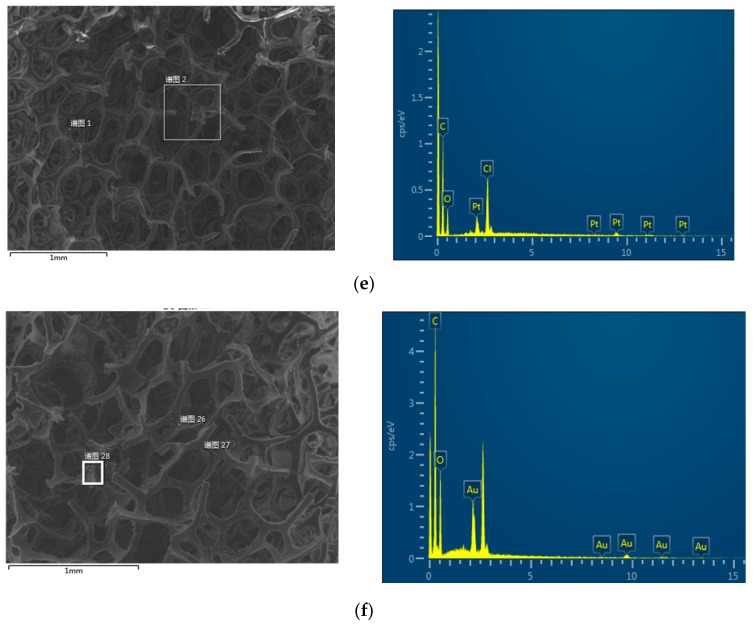
SEM image and energy-dispersive X-ray (EDX) analysis results of (**a**) Cu^2+^-, (**b**) Pb^2+^-, (**c**) Cd^2+^-loaded DTC-g-PE-DA@GB@PU and (**d**) Pd^2^^+^-, (**b**) Pt^4+^-, (**f**) Au^3+^-loaded PE-DA@GB@PU.

The mechanisms of HM and NM ions adsorption by DTC-g-PE-DA@GB@PU and PE-DA@GB@PU were also elucidated by the SEM/EDS measurements before and after loading the HM and NM ions, respectively. DTC-g-PE-DA@GB@PU and PE-DA@GB@PU were shaken in 100-ppm HM ions and 1000-ppm NM ions, respectively, for half an hour. The HM and NM ions were prepared using their nitrates and adjusted to optimal pH. The presence of Cu, Pb, Cd, Pd, Pt, and Au peaks, along with other elemental peaks (where the carbon (C) and oxygen (O) peaks were due to DTC-g-PE-DA@GB@PU and PE-DA@GB@PU) in the EDX analysis spectrum (Figure 8) confirm the adsorption of Cu^2+^, Pb^2+^, Cd^2+^ and Pd^2+^, Pt^4+^, Au^3+^ onto the surfaces of DTC-g-PE-DA@GB@PU and PE-DA@GB@PU, respectively [33,34].

**Figure 9:**
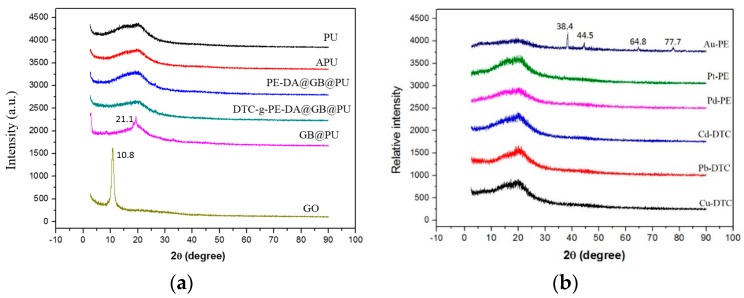
WAXD patterns for (**a**) PU, APU, GB@PU, PE-DA@GB@PU, DTC-g-PE-DA@GB@PU, and GO; (**b**) after Cu^2+^, Pb^2+^, and Cd^2+^ adsorption on the DTC-g-PE-DA@GB@PU and Pd^2+^, Pt^4+^, and Au^3+^ adsorption on the PE-DA@GB@PU.

The samples were subjected to WAXD analysis using a PANalytical diffractometer with Cu-Kα radiation (40 kV, 40 mA). The WAXD diffraction patterns of PU, APU, GB@PU, PE-DA@GB@PU, DTC-g-PE-DA@GB@PU, and GO are shown in Figure 9a. The WAXD diffraction pattern of PU exhibited a characteristic broad peak. GO shows a diffraction peak at ~10.8° [20]. After reaction of GO with APU, the diffraction peak of GB@PU was broadened and red shifted to 21.1°, compared with GO, suggesting a reduction in the content of oxygen-containing groups from GO to graphene [35]. For PE-DA@GB@PU and DTC-g-PE-DA@GB@PU, broad and weak peaks were observed in the WAXD patterns, corresponding to the amorphous feature of the crosslinked polymer matrix, implying the presence of intercalated PEI and PDA with GB@PU [36]. To explore the adsorption mechanism, DTC-g-PE-DA@GB@PU was added to copper nitrate, lead nitrate, and cadmium nitrate solution (HM ions ~1000 ppm) separately. Unlike the observations reported by Sharma et al. [33,34], no precipitate or floc appeared. We speculate that nitro-oxidized carboxycellulose nanofibers (NOCNF) constituted a kind of particle material, whereas the DTC-g-PE-DA@GB@PU was bulk material. The surface of the former was negatively charged, which could be induced to aggregate in the presence of cations. However, the form of DTC-g-PE-DA@GB@PU was bulk, and its adsorption characteristics were mainly due to its porous characteristics. In addition, the pH of the HM ion solutions may not be optimum for adsorption because the pH levels of 1000-ppm solutions of Cu^2+^, Pb^2+^, and Cd^2+^ were 4.04, 4.05, and 4.87, respectively. However, when some NaOH solution was added to adjust the pH to 6, precipitation appeared (Figure S9, Supplementary Materials). Then we reduced the concentration of the solutions to 100 ppm, and WAXD analysis of DTC-g-PE-DA@GB@PU was performed after adsorption (Figure 9b). Unfortunately, the WAXD patterns show no significant changes. The probable main reason was maybe that the low concentration of HM ion solutions resulted in low adsorption amount and the higher baseline of DTC-g-PE-DA@GB@PU masked the peaks of Cu, Pb, and Cd loaded on the adsorbent. For Pd^2+^, Pt^4+^, and Au^3+^, 1000-ppm solutions were used, because at low pH (pH = 0), no precipitation was observed. As shown in Figure 9b, after the impregnation of PE-DA@GB@PU into gold solution, new peaks appeared at 20 = 38.4°, 44.5°, 64.8°, and 77.7°, which matched exactly with those of gold, verifying the formation of elemental gold during the adsorption process. Chloroauric acid has high oxidation. Amino groups, which are the major surface constituents of PE-DA@GB@PU, can be easily reduced by AuCl_4_^−^ [37]. For Pd and Pt, there were no obvious changes in the WAXD patterns because the main adsorption mechanism may have been electrostatic attraction. In Figure 8d,e the EDX show the presence of chlorine atom, which could reinforce this assumption.

### 3.6. Regeneration of DTC-g-PE-DA@GB@PU and PE-DA@GB@PU

**Figure 10:**
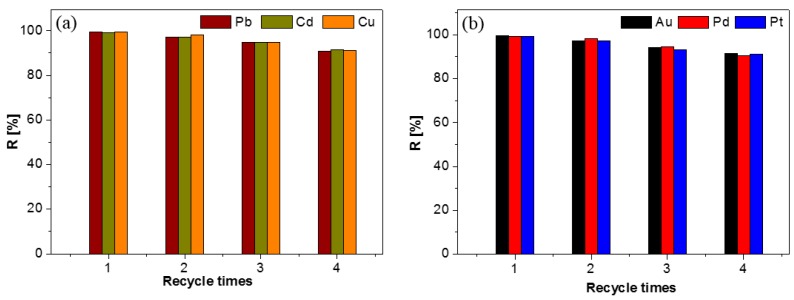
Recyclability of (**a**) DTC-g-PE-DA@GB@PU toward 10.0 μg mL^−1^ Cu^2+^, Pb^2+^ and Cd^2+^. Other conditions: solution pH = 6; volume, 20 mL; adsorbent dose, 20 mg; temperature, 25 °C; (**b**) recyclability of PE-DA@GB@PU toward 1.0 μg mL^−1^ Pd^2+^, Pt^4+^, and Au^3+^. Other conditions: solution pH = 0; volume, 20 mL; adsorbent dose, 20 mg; temperature, 25 °C.

Regeneration of adsorbents is essential in practical applications because of the economic and ecological appeals for sustainability. Thus, the adsorption–desorption ability of DTC-g-PE-DA@GB@PU was examined with of 0.1 M HCl and NaOH solutions. Both of these eluents were effective for leaching the HM ions. However, DTC-g-PE-DA@GB@PU decomposed under strong acid conditions. Therefore, when simple recovery of HM ions is desired, 0.1 M NaOH should be used as the eluent. After the adsorption of the HM ions, DTC-g-PE-DA@GB@PU was soaked in 0.1 M NaOH to remove the adsorbed HM ions. The adsorbent was then dried in air and the adsorption recycling was repeated again. The adsorption–elution processes were repeated for four successive cycles without evident loss of the adsorption capacity (Figure 10a). After four adsorption–desorption processes of using DTC-g-PE-DA@GB@PU to adsorb HM ions, 0.1 M HCl was used in the final desorption step to remove the HM ions. Simultaneously, PE-DA@GB@PU was obtained, which could then be used to directly recover NM ions. After the adsorption of NM ions by PE-DA@GB@PU, the sorbent was soaked in 1.0 M HNO_3_ to effectively remove the NM ions. In a similar manner as for DTC-g-PE-DA@GB@PU, the PE-DA@GB@PU sorbent was dried in air, and the adsorption cycle was repeated again. The extraction percentages of NM ions for PE-DA@GB@PU lost nearly 10% of the original efficiency after four cycles (Figure 10b).

The described method was also applied for the removal of Cu^2+^, Pb^2+^ and Cd^2+^ from industrial effluents and recovery of Pd^2+^, Pt^4+^, and Au^3+^ present in copper leaching residue and anode slime, successively. The industrial effluents and the refining wastes (copper leaching residue and anode slime) were obtained from Jinchuan Group International Resources Co. Ltd. The industrial effluents and the refining wastes (copper leaching residue and anode slime) produced in the refining process contained a certain amount of HMs and NMs, respectively. Sorption with these new adsorbents that allowed HM and NM deep concentration and their separation from other base metals proved to be one of the most efficient ways to recover HMs and NMs. Industrial effluents were adjusted to the desired pH values before the adsorption process. After the adsorption of HM ions, 0.1 M HCl was used as an eluent and PE-DA@GB@PU was obtained, which was then used to recover NMs from the copper leaching residue and anode slime. Before the recovery process, copper leaching residue and anode slime were treated with aqua regia by Elci’s procedure [38]. The method developed for the determination of HM and NM ions in industrial effluents, copper leaching residue, and anode slime from Jinchuan Group International Resources Co. Ltd. is summarized in Table 3.

## 4. Conclusions

Novel PU composites were synthesized and their adsorptive selectivities for HM and NM ions were investigated. The ‘S’ donor ligands of DTC-g-PE-DA@GB@PU were responsible for its high selectivity for Cu^2+^, Pb^2+^ and Cd^2+^; while PE-DA@GB@PU possessed abundant amine groups that obviously increased its adsorption capacity, and the composite exhibited excellent selectivity properties toward Pd^2+^, Pt^4+^, and Au^3+^. The adsorption processes depended on the pH value, contact time, and initial metal ion concentration. The optimal conditions, adsorption isotherms, and kinetics for recovery were investigated systematically. The proposed method was applied for the removal of HMs from industrial effluents and recovery of NMs from the copper leaching residue and anode slime, with satisfactory results being obtained in all cases.

## Figures and Tables

**Table 1:** Parameters of the Langmuir, Freundlich, and Dubinin–Radushkevich isotherms for the adsorption of HM and NM ions onto DTC-g-PE-DA@GB@PU and PE-DA@GB@PU, respectively, at 25 °C.

Isotherms	Isotherm Constants	Heavy Metal Ions			Noble Metal Ions		
		Cu^2+^	Pb^2+^	Cd^2+^	Pd^2+^	Pt^4+^	Au^3+^
Langmuir	*F*_max_ (mg g^−1^)	41.2	113.9	57.1	285.7	185.2	384.6
	*K* (L mg^−1^)	0.02915	0.3776	0.1726	0.0986	0.1192	0.2524
	*R* ^2^	0.9164	0.9935	0.9971	0.9985	0.9988	0.9997
	*R_L_*	0.2554–0.6317	0.0145–0.1169	0.0461–0.2246	0.0125–0.9103	0.0138–0.8935	0.0049–0.7985
Freundlich	*K_f_* (mg g^−1^) (L mg^−1^)^1/n^	2.816	44.17	15.57	37.025	32.946	58.844
	*n*	1.823	4.200	3.207	2.682	3.095	2.597
	*R* ^2^	0.9431	0.9891	0.9635	0.9653	0.9614	0.9102
Dubinin–Radushkevich	*F*_D-R_ (mg g^−1^)	30.6	98.2	45.6	128.14	101.64	195.43
	*E*	0.082	0.780	0.728	2.946	3.402	2.884
	*R* ^2^	0.9832	0.7024	0.8152	0.6336	0.7531	0.7947

**Table 2:** Kinetic parameters for the adsorption of HM and NM ions onto DTC-g-PE-DA@GB@PU and PE-DA@GB@PU, respectively (25 °C).

Model	Parameters	Heavy Metal Ions			Noble Metal Ions		
		Cu^2+^	Pb^2+^	Cd^2+^	Pd^2+^	Pt^4+^	Au^3+^
Pseudo-first-order	*k*_1_ (min^−1^) *R*^2^	0.1020 0.9440	0.1105 0.9649	0.1021 0.9657	0.1951 0.9456	0.2077 0.9240	0.1967 0.6513
Pseudo-second-order	*k*_2_ (g mg^−1^min^−1^) *R*^2^	0.6965 0.9988	2.2519 0.9995	1.1523 0.9993	5.1564 0.9997	1.1750 0.9838	26.8477 0.9998
Intraparticle diffusion	*K_i_* (mg g^−1^ min^−0.5^) *R*^2^	0.02166 0.8860	0.01451 0.7155	0.01773 0.8487	0.0247 0.6995	0.0369 0.7628	0.0118 0.3221

**Table 3:** Standard addition method for HM ions and NM ions determination in industrial effluents and copper leaching residue, anode slime, respectively.

Sample	Analytes	Added (μg g^−1^)	Found ^a^ (μg g^−1^)	Recovery (%)
Industrial effluents	Cu	—	112.6 ± 5.8	—
		100	209.2 ± 7.6	96.6
	Pb	—	12.7 ± 2.0	—
		10	22.6 ± 2.6	96.0
	Cd	—	5.4 ± 0.4	—
		10	14.6 ± 1.8	92.0
Copper leaching residue	Pd	—	13.2 ± 1.8	—
		20	32.1 ± 3.4	94.5
	Pt	—	20.0 ± 3.7	—
		20	40.7 ± 4.0	104
	Au	—	25.3 ± 2.4	—
		20	46.5 ± 3.0	106
Anode slime	Pd	—	33.4 ± 2.8	—
		30	62.1 ± 2.4	95.7
	Pt	—	8.3 ± 1.6	—
		10	17.7 ± 2.2	94.0
	Au	—	179.8 ± 3.5	—
		100	285.1 ± 2.2	105

^a^ Mean value ± standard deviation, *n* = 3.

## Supplementary Materials

The following are available online at https://www.mdpi.com/2073-4360/11/7/1125/s1, Figure S1: Raman spectra of PU, APU, GO, GB@PU, PE-DA@GB@PU and DTC-g-PE-DA@GB@PU, Figure S2: The acid decomposition of dithiocarbamate compound, Figure S3: The Langmuir sorption isotherm for batch method of (a) DTC-g-PE-DA@GB@PU for HM ions and (b) PE-DA@GB@PU for NM ions; (25 °C), Figure S4: The Freundlich sorption isotherm for batch method of (a) DTC-g-PE-DA@GB@PU for HM ions and (b) PE-DA@GB@PU for NM ions; (25 °C), Figure S5: The D–R sorption sorption isotherm for batch method of(a) DTC-g-PE-DA@GB@PU for HM ions and (b) PE-DA@GB@PU for NM ions; (25 °C), Figure S6: Pseudo-first-order plots for (a) HM ions on DTC-g- PE-DA@GB@PU and (b) NM ions on PE-DA@GB@PU at 25 °C, Figure S7: Pseudo-second-order plots for (a) HM ions on DTC-g-PE-DA@GB@PU and (b) NM ions on PE-DA@GB@PU at 25 °C, Figure S8: Intraparticle diffusion plots for (a) HM ions on DTC-g-PE-DA@GB@PU and (b) NM ions on PE-DA@GB@PU at 25 °C, Figure S9: The pH levels of 1000-ppm solutions of Cu^2+^, Pb^2+^, and Cd^2+^ after some NaOH solution was added to adjust the pH to 6, Table S1: Operation parameters of IRIS Advantage ICP-OES, Table S2: Comparison of the maximum adsorption capacities of DTC-g-PE-DA@GB@PU and PE-DA@GB@PU with other adsorbents.
