# Supplementary material for: Sequential Recovery of Heavy and Noble Metals by Mussel-Inspired Polydopamine-Polyethyleneimine Conjugated Polyurethane Composite Bearing Dithiocarbamate Moieties
Source: Polymers (Basel). 2019 Jul 2;11(7):1125. doi: 10.3390/polym11071125 (PMC6680459; doi:10.3390/polym11071125)
Supplement: Supplementary file 1 [file polymers-11-01125-s001.pdf]

Supporting Information

Sequential recovery of heavy metals and precious metals by mussel-inspired polydopamine-polyethyleneimine conjugated graphene-polyurethane composite bearing dithiocarbamate moieties

Dingshuai Xue [1,*], Ting Li [2], Guoju Chen [3], Yanhong Liu [1], Danping Zhang [1], Qian Guo [1], Jujie Guo [1], Yueheng Yang [1], Jiefang Sun [2], Benxun Su [1] Lei Sun [5] and Bing Shao [2]

[1] State Key Laboratory of Lithospheric Evolution, Institute of Geology and Geophysics, Chinese Academy of Sciences, Beijing 100029, China.
[2] School of Public Health, Capital Medical University, Beijing 100069, China.
[3] State Key Laboratory for Comprehensive Utilization of Nickel and Cobalt Resources, Jinchang, Gansu 737100, China.
[4] Beijing Key Laboratory of Diagnostic and Traceability Technologies for Food poisoning, Beijing Center for Disease Prevention and Control, 100013, China.
[5] Center for Biological Imaging, Institute of Biophysics, Chinese Academy of Sciences, Beijing 100101, China.
* Correspondence: xuedingshuai@mail.iggcas.ac.cn; Tel.: +86-10-82998487

The theoretical basis of the sorption isotherms and kinetic models

The experimental adsorption data were compared with the Langmuir, Freundlich, and Dubinin–Radushkevich (D–R) isotherm models. The Langmuir model is expressed as

$$D_e/F_e = D_e/F_{max} + 1/KF_{max} \quad (1)$$

where D_e is the equilibrium concentration of HM and NM ions in the solution (mg g^{-1}), F_e is the adsorption capacity of HM and NM ions adsorbed at equilibrium (mg g^{-1}), F_{max} is the theoretical maximum adsorption capacity (mg g^{-1}), and K is the Langmuir constant related to the affinity of the binding sites (L mg^{-1}). The slope and intercept of the plots of D_e/F_e versus D_e were used to calculate constants K and F_{max}. The Langmuir equation was analyzed using a dimensionless equilibrium parameter, R_L, which is also known as the separation factor. This parameter was used to evaluate whether the influence of the adsorption isotherm is favorable or unfavorable, and it can be expressed by the following equation:

$$R_L = \frac{1}{1+KD_i} \quad (2)$$

where K is the Langmuir constant (L mol^{-1}), and D_0 is the initial concentration (mol L^{-1}). $R_L > 1$ denotes unfavorable adsorption, $R_L = 1$ represents linear adsorption, and $R_L = 0$ indicates irreversible adsorption.

The Freundlich model can be applied for non-ideal sorption on heterogeneous surfaces and multilayer sorption. The linear form of the Freundlich isotherm model is represented as

$$logF_e = logK_f + \frac{1}{n}logD_e \quad (3)$$

where F_e and D_e have the same definitions as those in Eq. (1). K_f (mg g^{-1}) (L mg^{-1})$^{1/n}$ and n are the Freundlich constants related to adsorption capacity and adsorption intensity, respectively. The values of n and K_f were calculated from the slope and intercept of the plot of $log\ F_e$ versus $log\ D_e$.

The D–R isotherm was used to determine whether the adsorption processes are physical or chemical. The linearized D–R equation is expressed as

$$lnF_e = lnF_{D-R} - \beta\varepsilon^2 \quad (4)$$

where F_e is the adsorption capacity at equilibrium (mg g^{-1}), F_{D-R} is the maximum monolayer adsorption capacity (mg g^{-1}), β is the activity coefficient related to adsorption mean free energy (mol^2 J^{-2}), and ε is the Polanyi potential as represented by

$$\varepsilon = RTln(1 + \frac{1}{D_e}) \quad (5)$$

where R is the gas constant (J mol^{-1} K^{-1}), T is the absolute temperature (K), and D_e is the equilibrium concentration of the adsorbate in aqueous solution (mg L^{-1}). The mean free energy of adsorption E (kJ mol^{-1}) can be calculated using the previously determined coefficient β in accordance with

$$E = \frac{1}{\sqrt{-2\beta}} \quad (6)$$

The sorption energy E provides insight into whether the adsorption mechanism is physical or chemical. When $E < 8$ kJ/mol, physisorption may affect adsorption. If E is in the range of 8–16 kJ/mol, then adsorption is governed by chemisorption.

Pseudo-first-order, pseudo-second-order, and intraparticle diffusion kinetic models were applied. Eqs. (7)–(9) show the equations of the three kinetic models.
Pseudo-first-order kinetic model:

$$\log(F_e - F_t) = logF_e - \frac{k_1 t}{2.303} \qquad (7)$$

Pseudo-second-order kinetic model:

$$t/F_t = 1/k_2 F_e^2 + t/F_e \qquad (8)$$

Intraparticle diffusion kinetic model:

$$F_t = k_i \sqrt{t} \qquad (9)$$

where F_e and F_t (mg g^{-1}) are the amounts of HM and NM ions adsorbed at equilibrium and at time t, respectively; k_1 is the rate constant of pseudo-first-order adsorption (min^{-1}); k_2 is the pseudo-second-order adsorption rate constant (g mg^{-1} min^{-1}); and k_i is the intraparticle diffusion rate constant (mg g^{-1} min$^{-0.5}$).

Table S1. Operation parameters of IRIS Advantage ICP-OES.

Parameter	Value	
RF power (W)	1150	
Auxiliary gas flow rate (L min^{-1})	0.5	
Carrier gas flow rate (L min^{-1})	0.6	
Coolant gas flow rate (L min^{-1})	12	
Frequency of RF generator (MHz)	27.12	
Exposure time (s) Axial	Low (UV)	High (Visible)
	20	10
Analytical wavelength (nm)	Au 242.795	Pd 324.270
	Pt 265.945	Pb 220.353
	Cd 226.502	Cu 324.754

Table S2. Comparison of the maximum adsorption capacities of DTC-g-PE-DA@GB@PU and PE-DA@GB@PU with other adsorbents.

Adsorbent	Maximum adsorption capacity (mg/g)						Ref.
	Au	Pd	Pt	Cu	Pb	Cd	
Mesoporous carbons	492.5	63.6	78.0	—	—	—	[1]
DPTH-magnetic nanoparticles	6.2	7.7	0.4	—	—	—	[2]
Functionalized vinylbenzyl chloride–acrylonitryle–divinylbenzene copolymers bearing amino and guanidine ligands	190.0	280.0	245.0	—	—	—	[3]
L-lysine-modified crosslinked chitosan resin	70.3	109.5	129.3	—	—	—	[4]
Polyethyleneimine algal-based beads	—	136.2	115.1	—	—	—	[5]
Ethylenediamine modified persimmon tannin	1150.4	112.6	—	—	—	—	[6]
Polyethyleneimine modified core–shell type anion exchange resins	31.0	23.0	14.0	—	—	—	[7]
Cysteine modified silica gel	155.6	88.3	144.4	—	—	—	[8]
**PE-DA@GB@PU**	**384.6**	**285.7**	**185.2**	**—**	**—**	**—**	**Present work**
Sulfur-Functionalized Ordered Mesoporous Carbon	—	—	—	—	29.98	4.96	[9]
Meranti sawdust	—	—	—	32.1	34.2	—	[10]
PVA/graphene oxide nanofiber	—	—	—	32.4	—	44.9	[11]

Dithiocarbamate CNTs	—	—	—	101.5	—	202.4	[12]
Graphene oxide membrane	—	—	—	72.4	—	84.3	[13]
Carboxylated magnetic iron oxide nanoparticles	—	—	—	41.9	177.2	58.2	[14]
Chitosan/Sulfydryl-functionalized GO composite	—	—	—	235	226	117	[15]
Silica-supported dithiocarbamate adsorbent	—	—	—	20.3	70.4	40.5	[16]
Hydrogel-supported nanosized hydrous manganese dioxide				54.3	201.4	93.8	[17]
**DTC-g-PE-DA@GB@PU**				**28.7**	**113.9**	**57.1**	**Present work**

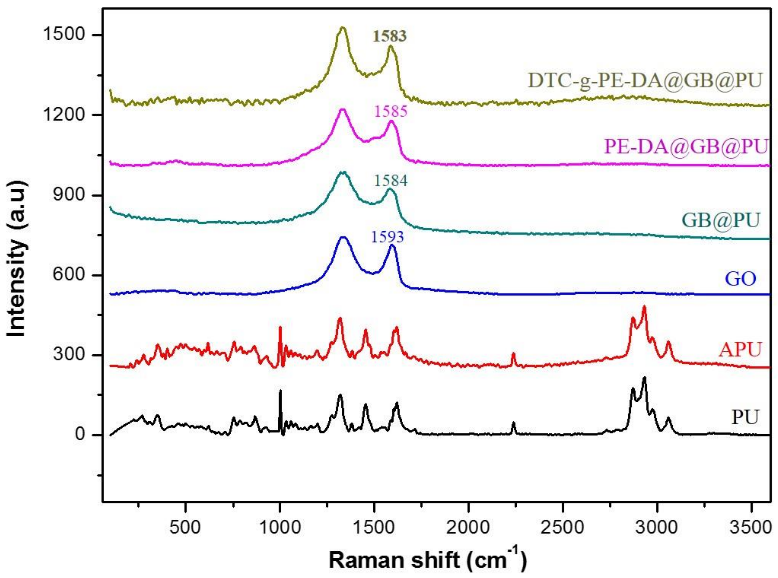

Figure S1. Raman spectra of PU, APU, GO, GB@PU, PE-DA@GB@PU and DTC-g-PE-DA@GB@PU.

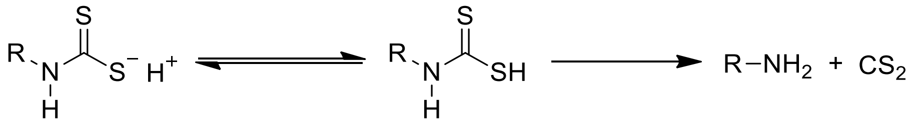

Figure S2. The acid decomposition of dithiocarbamate compound.

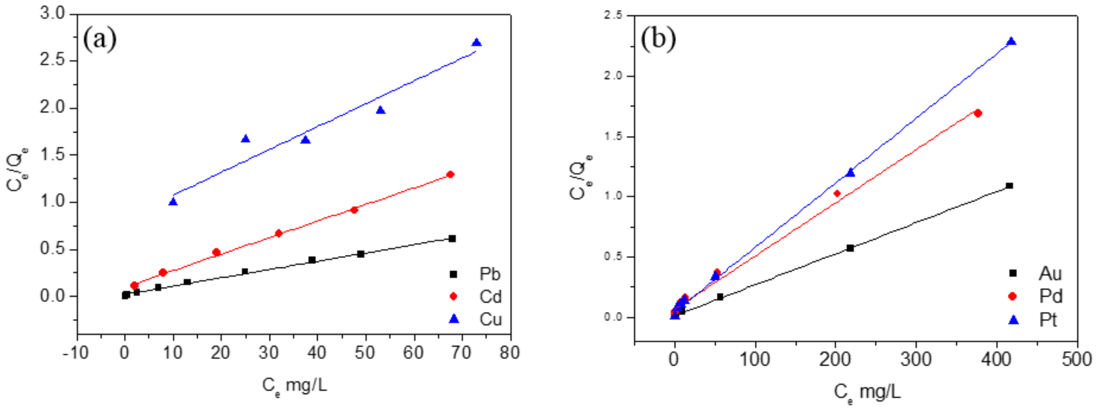

Figure S3. The Langmuir sorption isotherm for batch method of (a) DTC-g- PE-DA@GB@PU for HM ions and (b) PE-DA@GB@PU for NM ions; (25 °C).

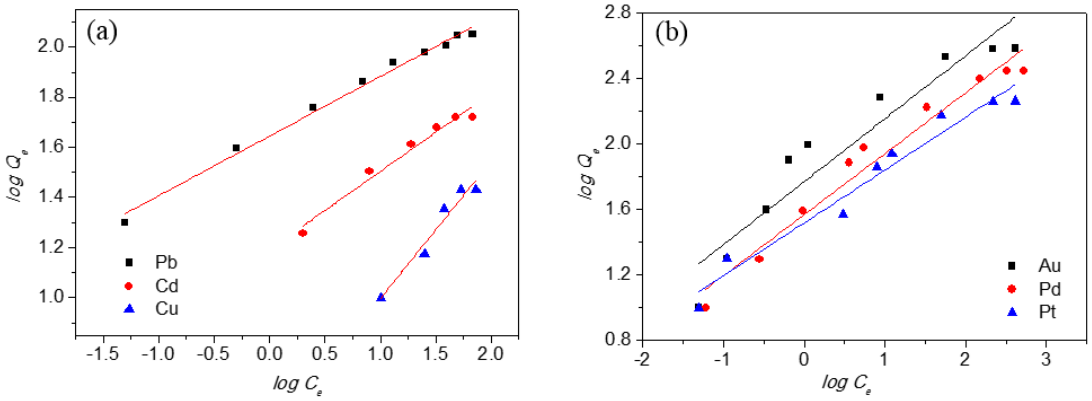

Figure S4. The Freundlich sorption isotherm for batch method of (a) DTC-g- PE-DA@GB@PU for HM ions and (b) PE-DA@GB@PU for NM ions; (25 °C).

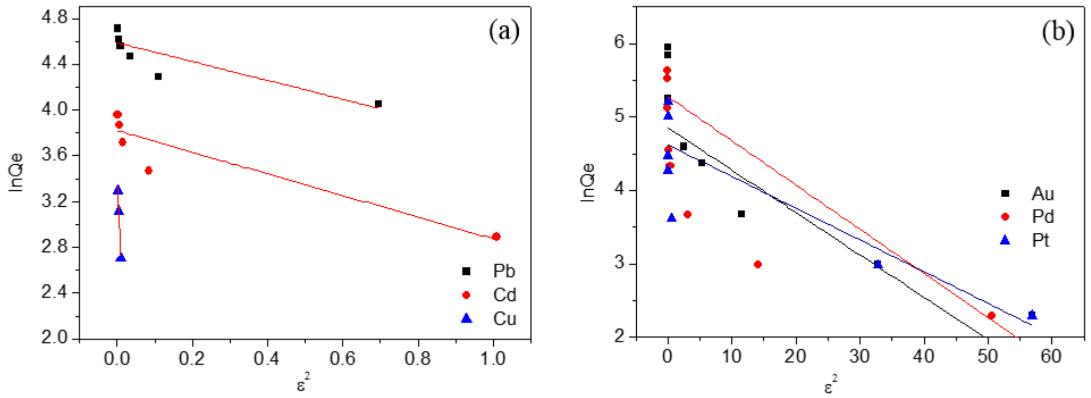

Figure S5. The D–R sorption sorption isotherm for batch method of(a) DTC-g- PE-DA@GB@PU for HM ions and (b) PE-DA@GB@PU for NM ions; (25 °C).

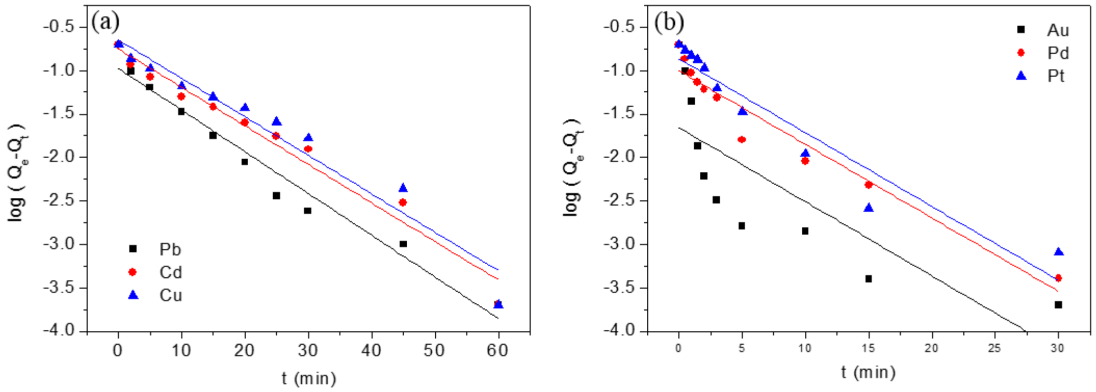

Figure S6. Pseudo-first-order plots for (a) HM ions on DTC-g- PE-DA@GB@PU and (b) NM ions on PE-DA@GB@PU at 25 °C.

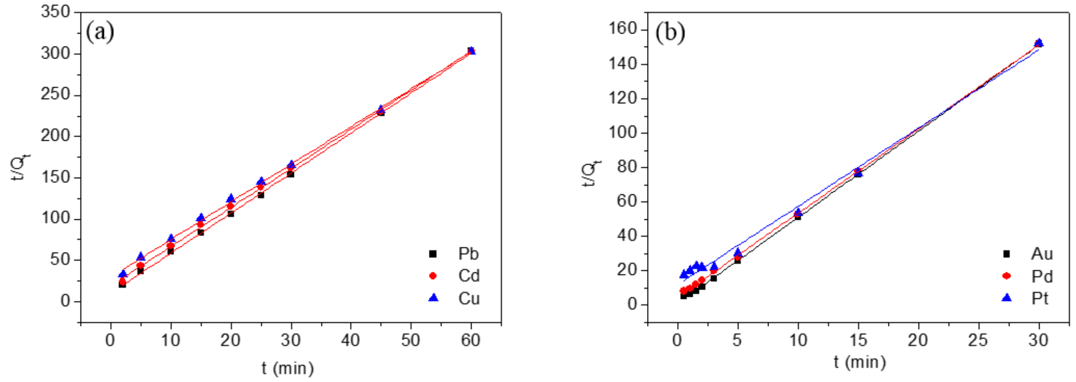

Figure S7. Pseudo-second-order plots for (a) HM ions on DTC-g- PE-DA@GB@PU and (b) NM ions on PE-DA@GB@PU at 25 °C.

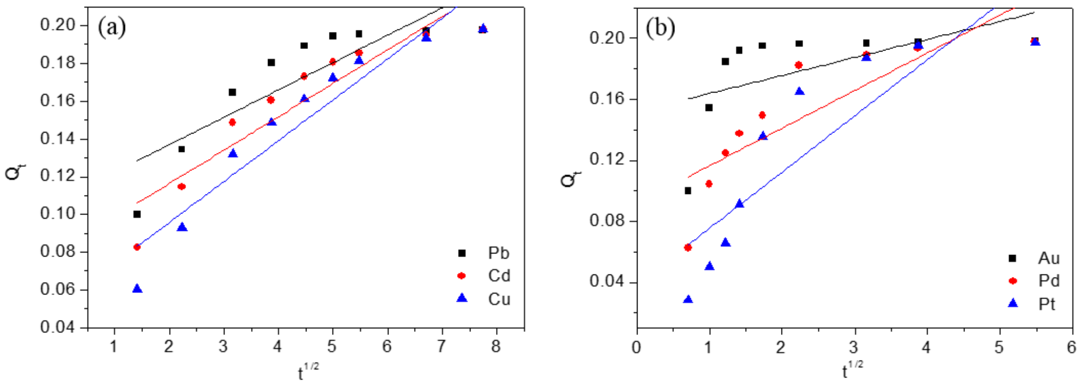

Figure S8. Intraparticle diffusion plots for (a) HM ions on DTC-g- PE-DA@GB@PU and (b) NM ions on PE-DA@GB@PU at 25 °C.

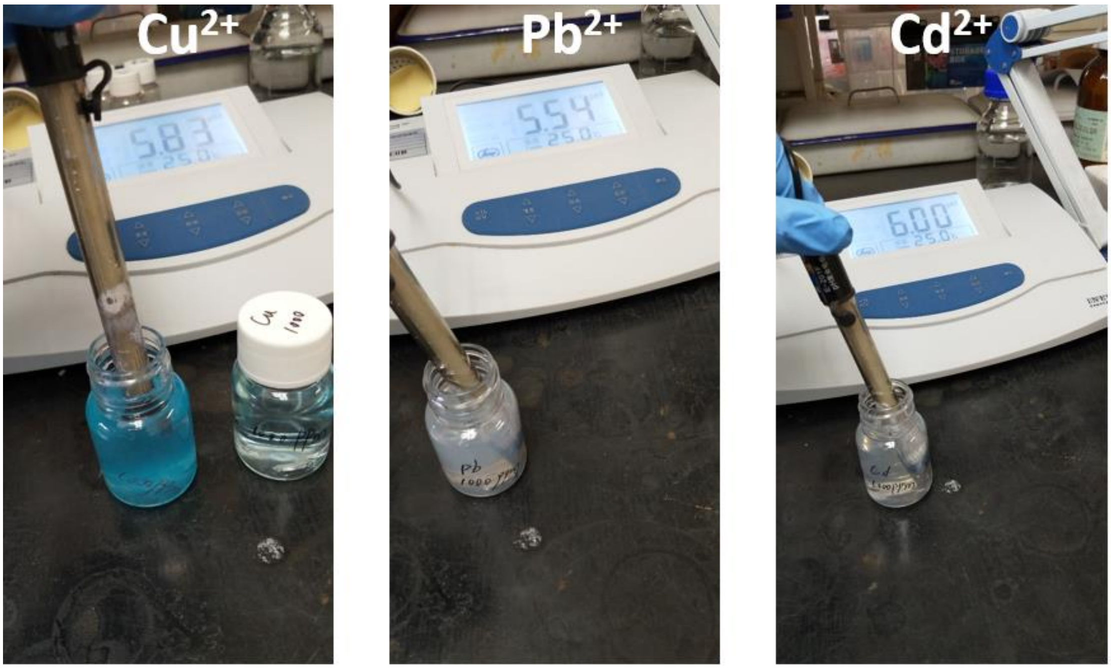

Figure S9. The pH levels of 1000-ppm solutions of Cu^{2+}, Pb^{2+}, and Cd^{2+} after some NaOH solution was added to adjust the pH to 6. (For Cu^{2+}, the color changed darker and blue precipitation appeared, while, for Pb^{2+}, and Cd^{2+}, white precipitation appeared. Pb^{2+} is easier to precipitate.)

Reference

[1] Zalupski, P. R., McDowell, R., Dutech, G. The Adsorption of Gold, Palladium, and Platinum from Acidic Chloride Solutions on Mesoporous Carbons. *Solvent Extraction and Ion Exchange* **2014**, *32*, 737-748.

[2] Guerrero, M. M. L., Alonso, E. V., García de Torres, A., Pavón, J. M. C. Simultaneous determination of traces of Pt, Pd, Os, Ir, Rh, Ag and Au metals by magnetic SPE ICP OES and in situ chemical vapour generation. *Journal of Analytical Atomic Spectrometry* **2017**, *32*, 2281-2291.

[3] Jermakowicz-Bartkowiak, D., Kolarz, B. N., Serwin, A. Sorption of precious metals from acid solutions by functionalised vinylbenzyl chloride–acrylonitryle–divinylbenzene copolymers bearing amino and guanidine ligands. *Reactive and Functional Polymers* **2005**, *65*, 135-142.

[4] Fujiwara, K., Ramesh, A., Maki, T., Hasegawa, H., Ueda, K. Adsorption of platinum (IV), palladium (II) and gold (III) from aqueous solutions onto L-lysine modified crosslinked chitosan resin. *Journal of hazardous materials* **2007**, *146*, 39-50.

[5] Wang, S., Vincent, T., Roux, J.-C., Faur, C., Guibal, E. Pd(II) and Pt(IV) sorption using alginate and algal-based beads. *Chemical Engineering Journal* **2017**, *313*, 567-579.

[6] Yi, Q., Fan, R., Xie, F., Min, H., Zhang, Q., Luo, Z. Selective Recovery of Au(III) and Pd(II) from Waste PCBs Using Ethylenediamine Modified Persimmon Tannin Adsorbent. *Procedia Environmental Sciences* **2016**, *31*, 185-194.

[7] Cyganowski, P., Jermakowicz-Bartkowiak, D. Synthesis and studies on core–shell type anion exchange resins based on a hybrid polymeric support. *Journal of Applied Polymer Science* **2016**, *133*.

[8] Mladenova, E., Dakova, I., Karadjova, I., Karadjov, M. Column solid phase extraction and determination of ultra-trace Au, Pd and Pt in environmental and geological samples. *Microchemical Journal* **2012**, *101*, 59-64.

[9] Saha, D., Barakat, S., Van Bramer, S. E., Nelson, K. A., Hensley, D. K., Chen, J. Noncompetitive and Competitive Adsorption of Heavy Metals in Sulfur-Functionalized Ordered Mesoporous Carbon. *ACS applied materials & interfaces* **2016**, *8*, 34132-34142.

[10] Rafatullah, M., Sulaiman, O., Hashim, R., Ahmad, A. Adsorption of copper (II), chromium (III), nickel (II) and lead (II) ions from aqueous solutions by meranti sawdust. *Journal of hazardous materials* **2009**, *170*, 969-977.

[11] Tan, P., Wen, J., Hu, Y., Tan, X. Adsorption of Cu2+ and Cd2+ from aqueous solution by novel electrospun poly(vinyl alcohol)/graphene oxide nanofibers. *RSC Advances* **2016**, *6*, 79641-79650.

[12] Li, Q., Yu, J., Zhou, F., Jiang, X. Synthesis and characterization of dithiocarbamate carbon nanotubes for the removal of heavy metal ions from aqueous solutions. *Colloids and Surfaces A: Physicochemical and Engineering Aspects* **2015**, *482*, 306-314.

[13] Tan, P., Sun, J., Hu, Y., Fang, Z., Bi, Q., Chen, Y., Cheng, J. Adsorption of Cu2+, Cd2+ and Ni2+ from aqueous single metal solutions on graphene oxide membranes. *Journal of hazardous materials* **2015**, *297*, 251-260.

[14] Xu, H., Yuan, H., Yu, J., Lin, S. Study on the competitive adsorption and correlational mechanism for heavy metal ions using the carboxylated magnetic iron oxide nanoparticles (MNPs-COOH) as efficient adsorbents. *Applied Surface Science* **2019**, *473*, 960-966.

[15] Li, X., Zhou, H., Wu, W., Wei, S., Xu, Y., Kuang, Y. Studies of heavy metal ion adsorption

on Chitosan/Sulfydryl-functionalized graphene oxide composites. *Journal of colloid and interface science* **2015**, *448*, 389-397.
[16] Bai, L., Hu, H., Fu, W., Wan, J., Cheng, X., Zhuge, L., Xiong, L., Chen, Q. Synthesis of a novel silica-supported dithiocarbamate adsorbent and its properties for the removal of heavy metal ions. *Journal of hazardous materials* **2011**, *195*, 261-75.
[17] Zhu, Q., Li, Z. Hydrogel-supported nanosized hydrous manganese dioxide: Synthesis, characterization, and adsorption behavior study for Pb2+, Cu2+, Cd2+ and Ni2+ removal from water. *Chemical Engineering Journal* **2015**, *281*, 69-80.
